# Exploration of Trends in Interspecific Abundance-Occupancy Relationships Using Empirically Derived Simulated Communities

**DOI:** 10.1371/journal.pone.0170816

**Published:** 2017-01-26

**Authors:** Christopher M. Martinez, Daniel E. Duplisea, Robert M. Cerrato, Michael G. Frisk

**Affiliations:** 1 School of Marine & Atmospheric Sciences, Stony Brook University, Stony Brook, New York, United States of America; 2 Fisheries and Oceans Canada, Institut Maurice-Lamontagne, Mont-Joli, Quebec, Canada; University of Saskatchewan, CANADA

## Abstract

The interspecific abundance-occupancy relationship (AOR) is a widely used tool that describes patterns of habitat utilization and, when evaluated over time, may be used to identify large-scale changes in community structure. Our primary goal for this research was to validate the utility of AORs as temporal indicators of community state. We used long-term survey data in four regions of the northwest Atlantic coastal shelf (NWACS) to estimate the diversity of spatial behaviors in each community, which we modeled with negative binomial (NB) distributions. NB parameters were used to generate time series data for simulated communities, from which AORs were then estimated and evaluated for temporal trends. We found that AORs from simulated communities were similar in year-to-year variation to empirical relationships. In order to further understand the role of spatial diversity in the generation of AOR trends, we did additional simulations where NB parameters were manually manipulated. In one instance, we ran simulations while holding species’ parameters constant over time. This treatment effectively removed trends, suggesting that temporal change in community relationships was the result of genuine variation in intraspecific spatial use. In another set of simulations, we conducted a case study to evaluate the impact of a select group of schooling and spatially aggregating species on an especially rapid shift in AORs in the Gulf of Maine from 1973 to 1983. Removals of these species reduced the magnitudes of most trends, demonstrating their importance to observed community changes. This research directly links variation in AORs to distribution and density-related processes and provides a potentially powerful framework to identify community-level change and to test ecological and mechanistic hypotheses.

## Introduction

The relationship between organism and habitat is among the most basic concerns in ecology. A common tool to assess patterns of habitat utilization is the abundance-occupancy relationship (AOR). AORs have been applied to a variety of biological systems, from terrestrial [1, 2], to marine [3], freshwater [4], and experimentally controlled protist communities [5]. In its interspecific form, the AOR is a collection of two measures for each species within a community; the proportion of all sampled locations where at least one individual was present (occupancy) and the number of individuals caught divided by either the total number of locations (global mean abundance, GMA) or by the number of occupied sites only (local mean abundance, LMA). The interspecific AOR has been used to investigate overriding patterns of spatial organization within communities, with its structure being attributed to factors such as species interactions [6], environmental/habitat change [7], niche breadth [8], and direct anthropogenic impacts [9]. When compared in a temporal framework, these methods capture important macroecological information that reflects transitory properties of biological communities and their environment. Therefore, AORs provide a useful tool to assess large-scale patterns and drivers of community structure.

A positive interspecific AOR has been cited as one of the most ubiquitous of ecological phenomena (e.g., [2, 10, 11]). However, the frequency of positive relationships may not be as compelling as sometimes portrayed. In fact, depending on the measure of abundance used (i.e., local or global abundance), the AOR may be constrained to be positive [12]. In such cases a null relationship is non-existent, even when species follow a Poisson distribution, displaying no aggregation [13]. Therefore, trying to isolate mechanisms can be a particularly dubious exercise, as their removal cannot completely eliminate the positive relationship. For example, Gaston and Warren [5] were unable to coerce significant changes in AORs or remove positive relationships in experimental microcosms exposed to a range of disturbance regimes. Understandably, this has led to varying opinions on the utility of AORs and on the presence of potential artifacts [13–17] as well as a general lack of consensus regarding mechanisms [18, 19].

Despite concerns, AORs have proved useful in a great number of studies in spatial ecology [19]. One application, which has only been studied in a few contexts, is the use of AORs to create indices of change for populations [20, 21], communities [9, 22], or both [3, 7]. In these studies, there is less emphasis on the generation or existence of a positive relationship itself, and more on temporal trends in AOR structure (although the two are certainly related).

Storch and Gaston [23] suggested that macroecological studies are useful for “untangling” complexity at lower levels of organization, as they encapsulate the emergent properties of effectively countless interactions. It follows then that to understand the interspecific AOR, one must start with the individual species that compose it. The link between levels of organization in AORs is straightforward: populations of different species compose observations in the interspecific AOR and any factor (whether biotic or abiotic) influencing a species’ abundance and distribution across a community will cause its location within the AOR to change, thereby impacting the collective structure of the community relationship (e.g., [3]).

The above framework suggests that the AOR necessarily relies on basic distributional properties of each population and may therefore be derived from first principles. Some researchers have posited that the structure of AORs, including the propensity for positive relationships, is expected when a collection of diverse spatial behaviors is assembled together [11, 13, 24]. Wilson [12] tested this idea with simulated population data that were generated from several statistical distributions and found that depending on the measure of abundance used, AORs could be positive, negative or zero. However, Webb *et al*. [17] questioned the relevance of Wilson’s methods to natural systems. In any case, reducing a community of organisms to its basic statistical components creates opportunities for direct hypothesis testing and also allows for one to address questions surrounding mechanistic forces underlying interspecific AORs.

Our study addresses concerns about the use of AORs as indicators of temporal community structure through methods designed to link AOR variation to fundamental processes impacting distribution and density. We developed a simulation approach to explore the ability of AORs to track spatial structure among four NWACS communities. Specifically, we fit negative binomial (NB) distributions to empirical catch data, which were then used to generate simulated communities that we evaluated with AORs. This allowed us to capture temporal changes in abundance and spatial aggregation over biologically realistic ranges, which to our knowledge, has rarely been done in the AOR literature. Our primary interest was to observe whether simulated communities displayed temporal trends comparable to those observed empirically. In other words, does realistic abundance-occupancy variation result from a collection of NB-distributed species? In addition, we applied simple manipulations to NB parameters to test specific questions regarding AORs. First, we tested whether AOR trends would occur in the absence of yearly variation in spatial behaviors (i.e., when species’ NB parameters were held constant with time). As this procedure retained interspecific spatial diversity within a given community, we expected to observe differences in AORs among regions, but not temporal trends within regions. Second, we focused on a period of particularly intense community change observed in the Gulf of Maine from 1973 to 1982 as a case study to explore how ecological processes may be manifested as trends in AORs. Here, we tested whether a rise in the abundance of lower trophic species that school or are known to aggregate, some of which may be linked to harvest removals of higher trophic species (e.g., [25]), was responsible for the observed AOR trend. We evaluated AORs with and without species with aggregating spatial tendencies. Here, we expected that simulations that included removals would display smaller AOR trends.

## Methods

### Empirical Data

We used bottom trawl data from the Northeast Fisheries Science Center’s (NEFSC) fall survey for all years available between 1963 and 2008. In order to avoid potential bias due to changes in the survey fleet [26], we did not to use data after 2008. The survey uses a depth-stratified, random sampling design where effort is proportional to strata area [27]. Data were divided among four contiguous regions that comprise much of the Northwest Atlantic Coastal Shelf (NWACS) ecosystem, and are largely accepted divisions based on physical topography and biological communities [28]. Regions (from north to south) were the Gulf of Maine (GOM), Georges Bank (GB), Southern New England (SNE) and the Mid-Atlantic Bight (MAB). For consistency, we used 30 species in each region to represent communities. Species’ inclusion was based on a ranked index (*S*) that reflected both the frequency of catch and the proportion of years encountered:

$$S=\frac{H_{i}}{H_{max}}+\frac{y_{i}}{y_{tot}}$$ (1)

The first term was the ratio of the total catch (i.e., number of individuals) in species *i* (*H*_*i*_) over all survey years relative to that of the most frequently caught species in the region (*H*_*max*_). The second term was the proportion of total surveyed years (*y*_*tot*_) that species *i* was caught. The purpose of this selection criterion was to define communities by a subset of 30 species that were abundant and prevalent across survey years. In doing so, this index also helped to exclude rare species that can be disproportionately underrepresented in surveys, leading to analytical artifacts (e.g., [29]). Within a region, the same 30 species were evaluated for all years present in order to maximize the number of years in which spatial behaviors were assessed and also to compare community states for a consistent assemblage of organisms.

### Species’ Statistical Distributions

We used the MASS package [30] in R [31] to estimate yearly negative binomial (NB) parameters for each species’ catches (i.e., counts of individuals) among sampled sites. The NB distribution was used because of its consideration of aggregation, an essential element in density-dependent ecological processes, and also due to its prevalence in wide-ranging investigations of distribution and spatial structure [11]. Its probability mass function is given by the equation:

$$p\left(x\right)=\left(\begin{array}{c} x+k-1\\ x \end{array}\right)p^{k}{\left(1-p\right)}^{x},$$ (2)

where *p* can be expressed in terms of mean (*μ*) and size (*k*)(Eq 3). The NB parameter *μ* describes species’ mean catches across sampled sites and the size parameter *k* is a measure of spatial aggregation. As *k* decreases, aggregation increases, and vice versa.

$$p=\left(\frac{k}{k+\mu }\right)$$ (3)

Substituting Eq 3 for *p* in Eq 2 yields the NB parameterization used in this study:

$$p\left(x\right)=\left(\begin{array}{c} x+k-1\\ x \end{array}\right){\left(\frac{k}{k+\mu }\right)}^{k}{\left(\frac{\mu }{k+\mu }\right)}^{x}.$$ (4)

For model-fitting purposes, we only included species in years where they were caught more than twice. We tested for overall changes in *μ* and *k* with time with the Mann-Kendall trend test. This analysis performs a rank correlation to assess whether a monotonic variable, here an NB parameter, changes over time [32]. The test produces a rank correlation coefficient, Kendall’s tau (τ), which describes the direction of the trend (increasing or decreasing) and the magnitude of temporal change.

### Monte Carlo Simulations

**Fig 1:**
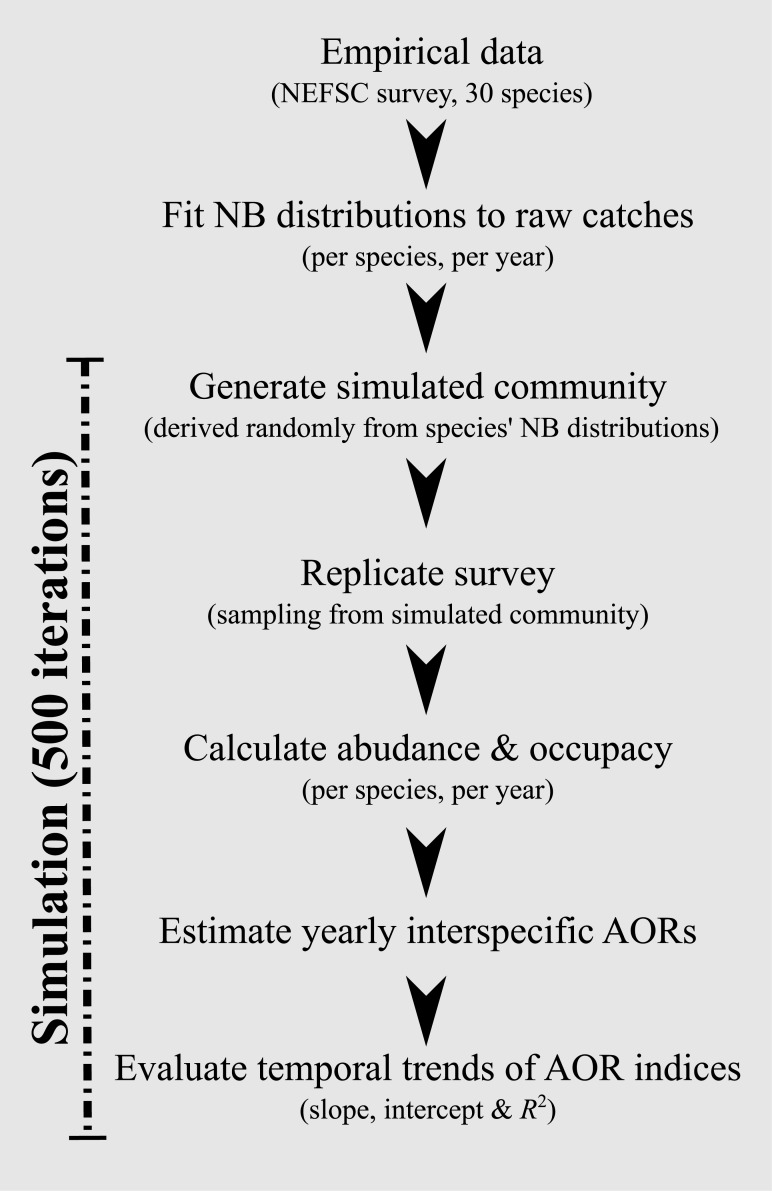
Outline of methods used to generate simulated communities and assess AOR trends.

We generated data for simulated communities, which consisted of 30 species whose abundances at each of 10,000 sites were random numbers drawn from their yearly estimated NB distributions (see Fig 1 for flow chart of methods). Based on previous unpublished work, we chose 10,000 sites in order to create a sufficiently large pool of values to sample from as to avoid potential biases due to unrealistically low variation in catches. Data for simulated communities were generated for all survey years, creating a full time series that reflected the temporally variable mix of distributions within natural communities. This routine was replicated for 500 Monte Carlo simulation runs.

We replicated a yearly trawl survey to sample from simulated communities by randomly selecting 55 sites per region, out of the 10,000 available. This level of sampling effort was well within the range of yearly trawls attempted for regional NEFSC fall surveys. Our approach assumes that species’ catches are representative of their true abundance, which is explicitly assumed whenever catches are used to infer relative abundance. We calculated interspecific occupancy values, local mean abundance (LMA), and global mean abundance (GMA) from the simulated and sampled data, as defined in the introduction. AORs were evaluated for each year using ordinary least-squares regression of arcsine transformed O on the natural logarithm of A (Eq 5), where *s* is the slope and *C* is the intercept [9].

O=sA+C (5)

Yearly variation in the AOR provided information on the manner by which communities collectively used available habitat and regression statistics (slopes, intercepts, and coefficients of determination, *R*^2^) were extracted to serve as indices of community state. Here, the linear model itself was not intended to infer a mechanism governing the relationship between abundance and occupancy; rather it was used to summarize the diversity of spatial behaviors exhibited by a community. Together, the AOR slope and intercept define the amount of habitat occupied by a community. The slope describes how occupied habitat is distributed among the organisms within a community. The presence of a significant slope (either negative or positive), suggests an inequality in the number of sites that species occupy. A positive slope means that low-abundance species are present in fewer sites than high-abundance species, a negative slope suggests the opposite to be true (low-abundance species occupy more sites), and a slope of zero means that all species occupy the same number of sites.

**Fig 2:**
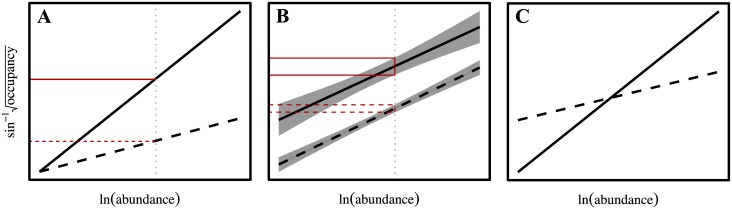
Forms of variation for interspecific AOR indices. Differences in slope (A) result in individuals occupying fewer sites (red horizontal lines) at a given abundance. Changes in AOR intercepts (B, regression lines) reflect a uniform shift in the total proportion of sites occupied. The *R*^2^ (B, shaded regions about regressions) describes the diversity of spatial occupancy strategies (red outlined boxes) utilized by species within a region. Often the slope and intercept both vary (C), and the degree to which each does, speaks to the specific nature of changes in habitat use across species.

Where slope signifies an inequality in habitat occupation among the species within a community, the AOR intercept sets the community-wide occupation of available habitat. All else being constant, changes in the intercept reflect uniform shifts in the total amount of available habitat occupied (i.e., the area under the regression line). Here, an increasing intercept means that all species are collectively occupying more habitat. As a consequence, larger intercepts result in lower density at a given level of abundance (vertical line in Fig 2B). For decreasing intercepts, the opposite is true. In reality, the AOR slope and intercept are often linked and in order to assess the nature of community variation, one must consider the interaction between the two. For example, Fig 2A shows regressions that vary with respect to slope, but have invariable intercepts. This constrains variation in low abundance species but amplifies it in the most abundant, such that overall community change is disproportionally concentrated in the latter. In comparison, AORs in which both slope an intercept change with time may reflect spatial changes occurring in both low and high abundance species. For this reason, it is also essential to assess community trends within the context of the internal dynamics of the species that compose them; the individual points in AORs.

Lastly, the AOR *R*^2^ may be thought of as an index of spatial heterogeneity of the species within each community. Communities with high *R*^2^ will have small variation with respect to spatial habitat occupation (Fig 2B, shaded region about dashed regression line). Conversely, low *R*^2^ values (Fig 2B, shaded region about solid regression line) indicate relatively large variance around predicted values, corresponding to a wider range of occupancy strategies. The use of AOR indices here assumes consistency in sampling across years and that species catchability due to gear selectivity does not change markedly.

For each simulation iteration we tested for evidence of overall temporal change in AOR indices using the Mann-Kendall trend test. Mean trends and 95% confidence intervals were calculated from simulation results. Significance of mean simulated trends was inferred if 95% confidence intervals excluded zero. Results were compared to empirical trends, in which AORs were calculated directly from catch data.

### NB Parameter Manipulations

Two additional sets of simulations were done in order to examine the influence of spatial diversity on overall community structure. The first of these tested whether a trend could be induced simply through stochastic year-to-year variation in spatially diverse assemblages where intraspecific variation remained constant over time. To do this, we determined each species’ median NB parameters over all years and ran simulations with these values held constant for the entire time series. The resulting AORs provided a direct contrast to simulations based on species with temporally variable NB distributions.

In order to give ecological context to an observed AOR trend, we used a second set of simulations as a case study that examined especially strong changes between 1973 and 1983 in the Gulf of Maine (GOM). During this time, a small subset of species with schooling/aggregation tendencies increased in relative abundance within the community and we wished to test whether they were driving observed changes in the AOR. In order to assess their relative impacts on community trends, we ran simulations with and without seven species that were identified as schooling or are otherwise known to occur in dense aggregations; Alewife (*Alosa pseudoharengus*), American shad (*Alosa sapidissima*), Atlantic argentine (*Argentina silus*), Atlantic herring (*Clupea harengus*), longfin squid (*Doryteuthis pealeii*), northern shortfin squid (*Illex illecebrosus*), and sea scallop (*Placopecten magellanicus*). Most of these, except for *P*. *magellanicus*, also tended to be secondary consumers or lower-level predators that would presumably benefit from intense commercial harvest that disproportionately impacted higher-level predators during this period [25].

## Results

### Temporal trends in NB parameters

Parameter estimates were variable with time and also among regions (S1 Fig). The two northernmost regions (GB and the GOM) displayed the strongest trends, each with significant temporal variation in both NB parameters (Table 1). In SNE, a significant trend was identified for *μ* but not for *k*. No trends were found for parameters in the MAB.

**Table 1:** Trends (Kendall's τ) of mean negative binomial (NB) parameter estimates, with *P*-values provided in parentheses. Regions included are the Gulf of Maine (GOM), Georges Bank (GB), Southern New England (SNE), and the Mid-Atlantic Bight (MAB).

region	size parameter (*k*)	mu parameter (*μ*)
**GOM**	**-0.22 (0.036)**	**0.52 (< 0.001)**
**GB**	**-0.38 (< 0.001)**	**0.50 (< 0.001)**
**SNE**	-0.040 (0.70)	**0.33 (< 0.01)**
**MAB**	-0.064 (0.56)	-0.17 (0.12)

### Temporal trends in AOR indices

Positive AORs were identified in all simulations evaluated with global mean abundance (GMA), meaning that a negative slope was never encountered in 90,000 possible instances, across all years, regions and simulation iterations. When local mean abundance (LMA) was used, only 11 (0.012%) negative AORs were found. Of these, 1 occurred in the GOM, 6 in GB, 1 in SNE, and 3 were in the MAB.

**Fig 3:**
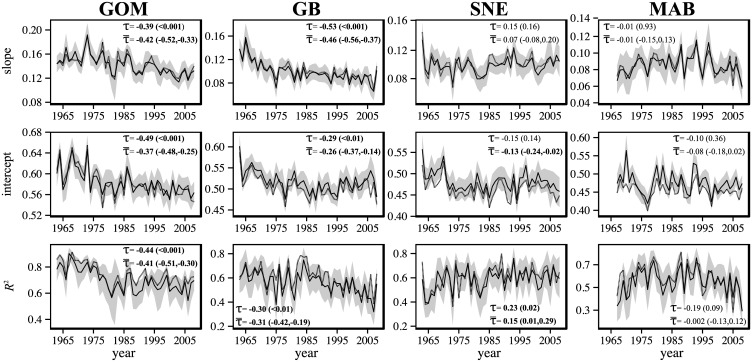
Mean of simulated AOR regression statistics (solid lines) for simulations based on global mean abundance (GMA). 95% confidence intervals on yearly values are shown in grey. For comparison, empirical estimates of AORs are provided (dashed lines). Trends (τ) of empirical indices are provided with *P*-values in parentheses as well as mean trends ($\bar{\tau }$) of simulated indices with 95% confidence intervals in brackets. Regions included are the Gulf of Maine (GOM), Georges Bank (GB), Southern New England (SNE), and the Mid-Atlantic Bight (MAB).

For GMA, simulated AOR indices displayed strong coherence with empirical AORs and temporal trends from simulations were of the same sign and of comparable magnitude as empirical results (Fig 3). Trends with time were strongest and most prevalent in northern regions. In the GOM, mean values of Kendall’s τ ($\bar{\tau }$) suggested significant trends for slope ($\bar{\tau }$ = -0.42, 95% CI [-0.52, -0.33]), intercept ($\bar{\tau }$ = -0.37, 95% CI [-0.48, -0.25]) and *R*^*2*^ ($\bar{\tau }$ = -0.41, 95% CI [-0.51, -0.30]). Similar results were found for GB slopes ($\bar{\tau }$ = -0.46, 95% CI [-0.56, -0.37]) intercepts ($\bar{\tau }$ = -0.26, 95% CI [-0.37, -0.14]) and *R*^2^ values ($\bar{\tau }$ = -0.31, 95% CI [-0.42, -0.19]). In SNE, a relatively small negative trend was identified for the AOR intercept, which just excluded zero from the 95% confidence interval ($\bar{\tau }$ = -0.13, 95% CI [-0.24, -0.02]). This was the only case where simulation and empirical results conflicted on the significance of a trend (the latter not being significant), although the magnitudes of the trends were still similar (Fig 3). The AOR *R*^2^ in SNE also displayed a small trend that excluded zero from the confidence interval ($\bar{\tau }$ = 0.15, 95% CI [0.01, 0.29]). These results suggest that the trend for *R*^2^ was in the opposite direction in SNE than in the GOM and GB, corresponding to an AOR that became stronger over time in the former. Lastly, there were no signs of overall trends for any AOR index in the MAB.

**Fig 4:**
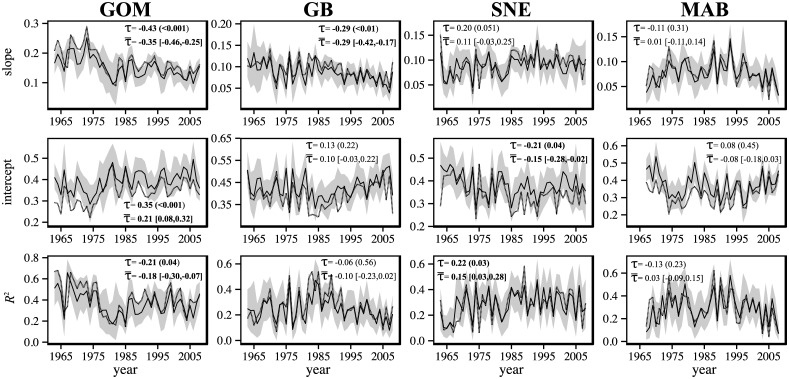
Mean of simulated AOR regression statistics (solid lines) for simulations based on local mean abundance (LMA). 95% confidence intervals on yearly values are shown in grey. For comparison, empirical estimates of AORs are provided (dashed lines). Trends (τ) of empirical indices are provided with *P*-values in parentheses as well as mean trends ($\bar{\tau }$) of simulated indices with 95% confidence intervals in brackets. Regions included are the Gulf of Maine (GOM), Georges Bank (GB), Southern New England (SNE), and the Mid-Atlantic Bight (MAB).

Simulated AORs based on LMA did not adhere to empirical results as well as GMA-based simulations (Fig 4). However, despite a few evident deviations, overall trends between simulations and empirical analyses were again comparable in direction and magnitude. Unlike GMA-based results, slope and *R*^2^ trends of LMA-based AORs shared directionality (i.e., both positive or both negative), with intercept trends always being opposite in direction (Fig 4). In the GOM, there was evidence for significant trends in slope ($\bar{\tau }$ = -0.35, 95% CI [-0.46, -0.25]), intercept ($\bar{\tau }$ = 0.21, 95% CI [0.08, 0.32]) and *R*^2^ ($\bar{\tau }$ = -0.18, 95% CI [-0.30, -0.07]). For the GB region, only slope had a significant trend over the time series ($\bar{\tau }$ = -0.29, 95% CI [-0.42, -0.17]), although the intercept and R^2^ both appeared to display directional change after 1985 (Fig 4). Trends were present for SNE intercepts ($\bar{\tau }$ = -0.15, 95% CI [-0.28, -0.02]) and *R*^2^ values ($\bar{\tau }$ = 0.15, 95% CI [0.03, 0.28]). Again, indices in the MAB showed no evidence of trends.

### Constant spatial behaviors

Simulations with constant, median parameters resulted in relationships that were temporally stable (S2 Fig), although variance around yearly means was still comparable to original simulations with variable NB parameters (Figs 3 and 4). Regional differences in interspecific composition were preserved, as indicated by different levels of mean index values maintained in each. Results for GMA and LMA were similar (S2 Fig), but as in original simulations, the latter often had larger variance around yearly mean AOR indices. Mean trends ($\bar{\tau }$) of indices were very small (S1 Table); often two or three orders of magnitude less than trends from simulations with temporally variable NB parameters. While 5% (± 0.34 SE) and 4.8% (± 0.20 SE) of iterations from GMA and LMA-based simulations (respectively) did yield significant trends, 95% confidence intervals were centered on a $\bar{\tau }$ of zero and clearly lacked directional change with time (S1 Table). The removal of trends through stabilization of species’ NB distributions indicates that those observed in original simulations were due to temporal changes in intraspecific habitat utilization.

### Impact of schooling/aggregating species

**Fig 5:**
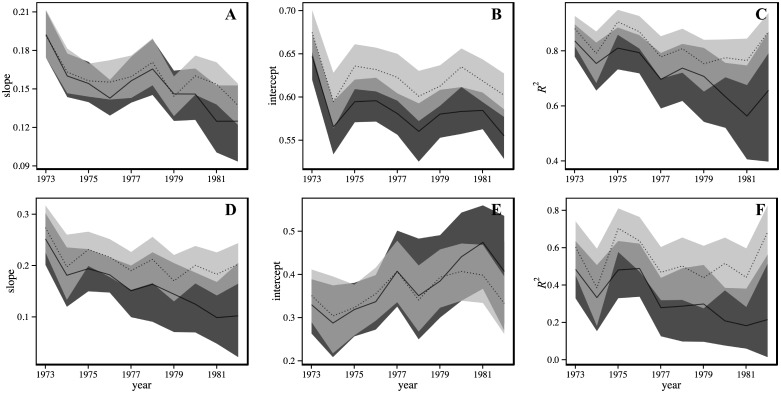
Mean AOR regression statistics for simulations in the Gulf of Maine (GOM) from 1973 to 1982. Results are given for relationships estimated with global mean abundance, GMA (A-C) and local mean abundance, LMA (D-F). Simulations including all species are shown in dark gray (solid line) and those with schooling and aggregating species removed are in light gray (dotted line). 95% confidence intervals are shown as shaded regions around yearly means.

Removals of seven aggregating species in the GOM from 1973 to 1982 caused a number of changes in AOR trends (Fig 5). Overall, the impacts of species removals were greater for LMA-based simulations than GMA-based. For GMA, the mean slope trend ($\bar{\tau }$) was slightly reduced from -0.55 (95% CI -0.78, -0.29) with all species included to -0.42 (95% CI -0.69–0.11) for species removals. Here, much of the difference in slopes between the two sets of simulations appeared to be concentrated in the last few years (Fig 5A). In contrast, for LMA relationships, divergence in slopes occurred much earlier and across much of the time series (Fig 5D). In this case, mean slope trends declined from -0.63 (95% CI -0.91, -0.33) to -0.37 (95% CI -0.69, -0.02).

GMA-based simulations also resulted in intercepts that differed over much of the time series, while LMA-based simulations had intercepts with differences concentrated in latter years (Fig 5B & 5E, respectively). For GMA, a fairly uniform shift to larger intercepts across years resulted in similar trends before and after removals ($\bar{\tau }$ = -0.33, 95% CI [-0.60, -0.02] versus $\bar{\tau }$ = -0.26, 95% CI [-0.56, -0.02]). In contrast, removals in LMA-based simulations caused a decrease of intercepts in 1980–1982 and trends were reduced by half, from $\bar{\tau }$ = 0.50 (95% CI 0.16, 0.80) to $\bar{\tau }$ = 0.25 (95% CI -0.07, 0.56). It is notable that a trend of zero was now included in the 95% confidence interval for simulations where species were removed.

Species removals had the largest impacts on *R*^2^ trends, where AORs became comparatively tighter (larger *R*^2^), especially in latter years (Fig 5C & 5F). GMA simulations saw a decline in trends from $\bar{\tau }$ = -0.56 (95% CI -0.82, -0.20) to $\bar{\tau }$ = -0.26 (95% CI -0.51, 0.02). A similar decrease was seen for LMA relationships ($\bar{\tau }$ = -0.49, 95% CI [-0.78, -0.11] to $\bar{\tau }$ = -0.07, 95% CI [-0.38, 0.20]). In both GMA and LMA, removals drove trends down such that zero was now included in 95% confidence intervals.

## Discussion

This research resulted in two key outcomes that further our understanding of AORs. First, simulated communities shared major patterns and features of variation with natural communities, validating empirical trends in the AOR by linking them to changes in species’ spatial distributions (NB parameters). Second, we showed that trends in AORs are sensitive to ecological processes. Here, a rapid shift in community structure in the GOM was a least partially attributed to a rise of schooling and aggregating species. Consequently, we suggest that the AOR is a suitable indicator of change at the community level, encompassing processes relating to assemblage structure, ecological interactions, and any other factor affecting habitat use.

Given that estimated NB distributions represented year-to-year spatial characteristics of species, we interpret the existence of trends in AOR indices to reflect genuine variation in the spatial diversity contained within communities and conclude that fundamental changes have occurred in patterns of habitat occupation in three of four regions investigated here (i.e., GOM, GB, and SNE). Our approach allowed for assessment of the reliability of trends through calculations of 95% confidence intervals from repeated simulation trials. This research supports the idea that the species that construct AORs may be thought of as a collection of spatial behaviors [11, 17, 24], in this case, represented by the negative binomial distribution.

For all sets of simulations, there were a number of differences for analyses based on global mean abundance (GMA) and local mean abundance (LMA). Which abundance measure an investigator should use depends on their research goals [17]. AORs estimated with GMA often have larger slopes and are less variable (higher *R*^2^), as indicated by their limitation to positive AORs. However, LMA is often regarded as a more ecologically relevant measure [19]. This may be, in part, because the generally larger variance of LMA-based AORs (i.e., smaller *R*^2^) and its ability to achieve negative relationships mean that disparate spatial strategies are more readily identified relative to GMA-based relationships (see Fig 2B in [17]).

Despite LMA relationships displaying some deviations between empirical and simulated results, their overall trends were still consistent; any time an empirical result was significant, zero was excluded from the 95% confidence intervals of simulated trends (Fig 4). For GMA-based results, only the AOR intercept in SNE showed a disagreement between empirical and simulated trends; the empirical trend was not significant (p = 0.14), but the 95% confidence interval for simulations just excluded a trend of zero (CI -0.24, -0.02). This aside, other minor differences between empirical and simulated results were not so marked as to affect overall interpretations. It is possible that discrepancies, when they occurred, may not fully be a failure of NB distributions to adhere to empirical variation, but can also be due to concessions that were necessary for parameter fitting (i.e., removal of species from years where they were not caught more than twice).

### Intraspecific Variation in Interspecific AORs

Holt *et al*. [11] questioned whether, “…spatial distribution strictly provides an explanation of interspecific occupancy-abundance relationships, rather than simply a rephrasing of one macroecological pattern in terms of another”. Simulation results presented herein suggest the former; that consideration of spatial distributions will likely play vital role in our eventual understanding of AOR mechanisms. As we have demonstrated through manipulations of NB parameters, the explicit link between spatial distributions and the AOR creates opportunities for rigorous hypothesis testing. The ability to eliminate trends in regional simulated communities by holding NB parameters constant over time confirmed that while a collection of spatial behaviors (i.e., interspecific variation) will recreate an AOR, yearly change is only achieved through intraspecific variation. These results suggest that in addition to the utility of the AOR to monitor community change, a simulation approach offers a framework from which researchers may test a number of ecological hypotheses based on different modes of NB manipulation.

### The Rise of Schoolers & Aggregators in the Gulf of Maine

The result of manual removals of aggregating species from AORs in the Gulf of Maine (GOM) between 1973 and 1982 was often a decrease in the magnitudes of trends in AOR indices (Fig 5), indicating that intraspecific changes in these species were important components in the observed community variation. The effect of removals on the AOR slope was larger for relationships estimated with LMA than with GMA (Fig 5A & 5D). This likely reflects the spatial ecology of removed species, where changes in aggregating species with patchy distributions will be more evident at local scales than regional. Furthermore, the existence of intercept trends in GMA simulations before and after species removals (Fig 5B) points to an additional source of decline in habitat occupation than the one tested here. The large decline in *R*^2^ trends (Fig 5C & 5F) suggests that as aggregating species increased in abundance, they added a new mode of spatial behavior to communities (i.e., high abundance and low occupancy), that did not otherwise exist in the remaining demersal species. It is not clear whether the observed rise of aggregating species (typically at lower trophic levels) in NWACS regions may be attributed to top-down effects of commercial harvest on higher trophic species (e.g., [25, 33–36]), but the results do show that our approach can be sensitive to the types of ecological processes associated with fishery-related assemblage disturbances.

### Future Applications & Directions

As a record of community state that may be tied to a broad range of ecological processes, AORs offer a great opportunity to assess the impacts of environmental variation. Currently, climate change is among the most scrutinized factors driving distributional shifts in marine organisms. Many studies take a Lagrangian approach, tracking parcels (populations) in space and relating the magnitude of displacement relative to some climate factor [37–40]. While such studies assess a major consequence of climate change, the spatial translation of populations, they exclude other density-dependent processes relating to in situ habitat utilization. AORs, on the other hand, explicitly model the organism-habitat relationship in a more comprehensive manner, that includes (but is not limited to) population movements. Therefore, temporal analyses of AORs may represent a complement to movement-focused studies to further our understanding of the impacts of climate change on communities.

Another prospective utility of temporal analyses of AORs is in the areas of conservation and resource management. AORs integrate a level of species’ interdependence that is consistent with advocated objectives of ecosystem-based management [41]. In a study on abundance-distribution relationships on the Scotian Shelf, Fisher and Frank [3] found a marked change in the slope of the relationship between 1970 and 2001, indicating a general increase in density within occupied habitat. The authors note that such a pattern was consistent with a well-known consequence of size-selective harvest strategies, decreasing body size, which reduces resource demands and allows more individuals to occupy a given area. In our study, AORs in the Gulf of Maine and Georges Bank, which are both adjacent to the western end of the Scotian Shelf, also displayed strong trends in slope that were consistent with patterns identified by Fisher and Frank [3]. Further work will be needed to confirm the relationship between AOR slopes and body size in the regions investigated here, but the potential for AORs to address such questions is promising.

The framework that we presented opens many opportunities for future research on community dynamics and the factors, both internal and external, which drive them. For example, one may imagine a simulation where NB parameters are allowed to vary based on expected species’ responses to changing levels of climate change or harvest. The experimental nature of these methods may also lead to a deeper understanding of mechanisms underlying AORs, as we have begun to show with the examples presented in this research.

## Supporting Information

S1 Table AOR regression statistics from simulations with NB parameters held constant. (PDF)

S1 Fig Time series of species’ negative binomial (NB) parameters. (PDF)

S2 Fig Plot of simulation results with NB parameters held constant. (PDF)

S1 File Metadata for codes used in S2 and S3 Files. (TXT)

S2 File Regional catch data used for analyses in this study. (CSV)

S3 File Yearly numbers of tows for regions included in this study. (CSV)

S4 File Sample R code for simulations and trend analyses in the Gulf of Maine. (TXT)
