# Supplementary material for: Exploration of Trends in Interspecific Abundance-Occupancy Relationships Using Empirically Derived Simulated Communities
Source: PLoS One. 2017 Jan 26;12(1):e0170816. doi: 10.1371/journal.pone.0170816 (PMC5268422; doi:10.1371/journal.pone.0170816)
Supplement: S1 Table — (PDF) [file pone.0170816.s001.pdf]

Table S1. Mean simulated trends for A-O relationships, estimated with constant NB parameters across all years. Results shown for simulations based on global mean abundance (GMA) and local mean abundance (LMA). Trends are shown with 95% confidence intervals. The percent of significant trends (out of 500 simulations) is also provided. Regions included are the Gulf of Maine (GOM), Georges Bank (GB), Southern New England (SNE) and the Mid-Atlantic Bight (MAB).

method	region	A-O index	mean $\tau_{simulated}$ (95% CI)	% sig.
**GMA**	**GOM**	slope	-0.0047 (-0.215, 0.190)	6.2
		intercept	-0.0014 (-0.196, 0.194)	4.0
		R^2	0.0097 (-0.169, 0.203)	3.8
	**GB**	slope	0.00042 (-0.200, 0.197)	4.4
		intercept	-0.0052 (-0.198, 0.202)	4.8
		R^2	0.0025 (-0.194, 0.194)	4.4
	**SNE**	slope	-0.00012 (-0.219, 0.206)	6.4
		intercept	0.0038 (-0.190, 0.209)	4.6
		R^2	-0.0012 (-0.192, 0.196)	4.4
	**MAB**	slope	0.0017 (-0.231, 0.216)	7.2
		intercept	0.0032 (-0.199, 0.210)	3.8
		R^2	0.0018 (-0.217, 0.209)	5.0
**LMA**	**GOM**	slope	-0.0048 (-0.213, 0.187)	5.8
		intercept	-0.0038 (-0.206, 0.183)	4.4
		R^2	-0.00041 (-0.211, 0.206)	5.8
	**GB**	slope	0.010 (-0.194, 0.189)	4.2
		intercept	0.0097 (-0.191, 0.195)	4.0
		R^2	0.010 (-0.195, 0.199)	4.8
	**SNE**	slope	0.00032 (-0.188, 0.204)	4.6
		intercept	-0.0011 (-0.197, 0.207)	4.4
		R^2	-0.00020 (-0.179, 0.206)	3.6
	**MAB**	slope	-0.0014 (-0.200, 0.215)	5.4
		intercept	-0.00062 (-0.215, 0.202)	5.2
		R^2	-0.00071 (-0.207, 0.210)	4.8
