# Supplementary material for: Exploration of Trends in Interspecific Abundance-Occupancy Relationships Using Empirically Derived Simulated Communities
Source: PLoS One. 2017 Jan 26;12(1):e0170816. doi: 10.1371/journal.pone.0170816 (PMC5268422; doi:10.1371/journal.pone.0170816)
Supplement: S1 Fig — (PDF) [file pone.0170816.s002.pdf]

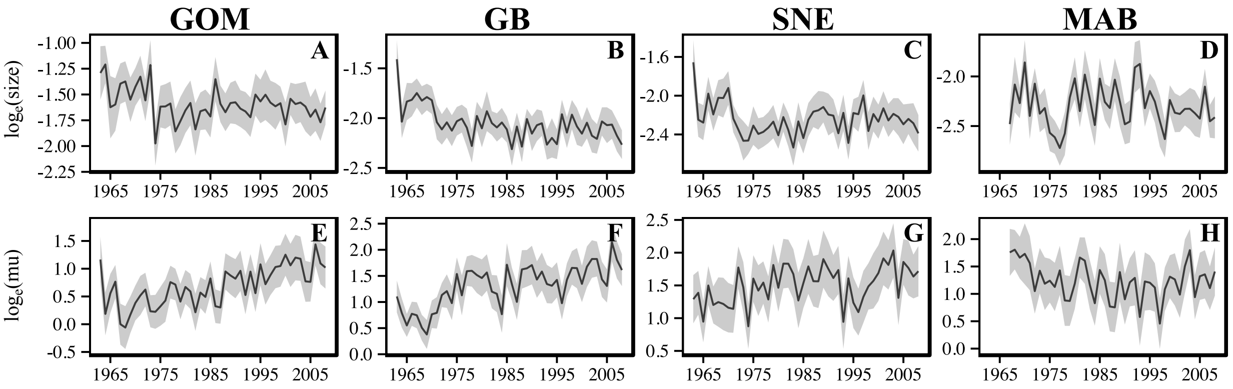

Figure S1. Time series of the natural log of mean negative binomial parameters (rows) for the Gulf of Maine (GOM), Georges Bank (GB), Southern New England (SNE), and the Mid-Atlantic Bight (MAB). Standard error is shown as the shaded gray region.
