# Supplementary material for: Exploration of Trends in Interspecific Abundance-Occupancy Relationships Using Empirically Derived Simulated Communities
Source: PLoS One. 2017 Jan 26;12(1):e0170816. doi: 10.1371/journal.pone.0170816 (PMC5268422; doi:10.1371/journal.pone.0170816)
Supplement: S2 Fig — (PDF) [file pone.0170816.s003.pdf]

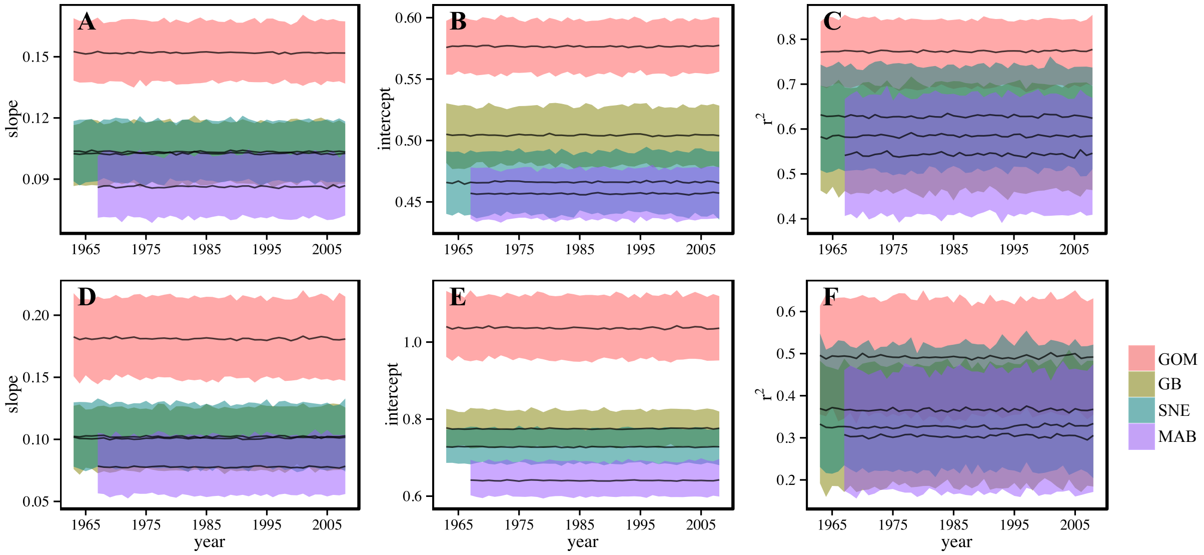

Figure S2. Mean AOR regression statistics for simulations with negative binomial (NB) parameters held constant. Results are given for relationships estimated with global mean abundance, GMA (A-C) and local mean abundance, LMA (D-F). In each plot, 95% confidence intervals are color-coded by region. Regions included are the Gulf of Maine (GOM), Georges Bank (GB), Southern New England (SNE), and the Mid-Atlantic Bight (MAB).
